# Fine Structure Investigation and Laser Cooling Study of the CdBr Molecule

**DOI:** 10.3390/ijms27010184

**Published:** 2025-12-23

**Authors:** Ali Mostafa, Israa Zeid, Nariman Abu El Kher, Nayla El-Kork, Mahmoud Korek

**Affiliations:** 1 Faculty of Science, Beirut Arab University, P.O. Box 11-5020 Riad El Solh, Beirut 1107 2809, Lebanonisraa.zeid93@gmail.com (I.Z.); mahmoud.korek@bau.edu.lb (M.K.); 2 Department of Physics, Khalifa University, Abu Dhabi P.O. Box 57, United Arab Emirates

**Keywords:** ab initio calculation, electronic structure, rovibrational calculation, dipole moments, Franck–Condon factor, radiative lifetime, laser cooling

## Abstract

The ab initio calculations of the electronic structure of the low-lying electronic states of the CdBr molecule are characterized in the ^2S+1^Λ^(+/−)^ and Ω^(+/−)^ representations using the complete active-space self-consistent field (CASSCF) method, followed by the multireference configuration interaction (MRCI) method with Davidson correction (+Q). The potential energy curves are investigated, and spectroscopic parameters (T_e_, R_e_, ω_e_, B_e_, $\alpha_{e}$, $\mu_{e}$, and D_e_) of the bound states are determined and analyzed. In addition, the rovibrational constants (E_v_, B_v_, D_v_, R_min_, and R_max_) are reported for the investigated states with and without spin–orbit coupling. The electronic transition dipole moment curve (TDMC) is obtained for the C^2^Π_1/2_ − X^2^Σ^+^_1/2_ transition. Based on these data, Franck–Condon factors (FCFs), Einstein coefficient of spontaneous emission A_ν’ν_, radiative lifetime τ, vibrational branching ratios, and the associated slowing distance are evaluated. The results indicated that CdBr is a promising candidate for direct laser cooling, and a feasible cooling scheme employing four pumping and repumping lasers in the ultraviolet region with suitable experimentally accessible parameters is presented. These findings provide practical guidance for experimental spectroscopists exploring ultracold diatomic molecules and their applications.

## 1. Introduction

Group IIB metal monohalides have attracted broad interest across various disciplines, including high-temperature chemistry [1] and studies of chemical reactions and environmental pollution [2,3]. Among these species, cadmium and zinc bromide radicals (CdBr and ZnBr) are particularly noteworthy due to their applications in metal–halide lasers [4]. Cadmium, a toxic 4d transition metal, exhibits long atmospheric residence times and undergoes extensive long-range transport from its emission sources, which are dominated by natural volcanic activity [5,6]. Human activities release various amounts of cadmium into the atmosphere that are similar to or exceed those from natural sources, primarily driven by the incineration of wastes and non-ferrous metals production [5]. A detailed understanding of cadmium’s gas phase chemistry is essential for clarifying the mechanisms of particulate cadmium formation and for providing a foundation for future investigations into its heterogeneous chemistry. Moreover, gas phase reactions between cadmium and halogen-containing species are significant in combustion processes, thereby contributing to understanding the primary pathways by which cadmium enters the atmosphere [2,3].

In recent years, the ability to cool molecular gases to ultralow temperatures has opened new frontiers in quantum dynamics, quantum chemistry, and many-body physics, enabling precise control over molecular motion and interactions [7,8,9,10]. Cold and ultracold molecules have emerged as versatile platforms for exploring fundamental physical phenomena [11,12] and for enabling practical applications, including precision measurements [13,14], quantum information processing [15], simulation of solid state models [16,17], and ultracold controlled chemistry [18], with additional emerging applications in nanolithography and Bose-stimulated chemistry [19]. Within this context, several metallic halide molecules [20,21,22,23,24] have been investigated both theoretically and experimentally for their potential in laser cooling. In particular, bromine-containing species often show well-resolved isotopic features and significant spin–orbit coupling in vibronic transitions [25]. Our group has previously explored the electronic structures of various group IIB monohalides, including ZnI [26], ZnX (X = F, Cl, Br, I) [27], ZnF [28], HgF [29], ZnBr [30], and CdX (X = F, Cl, Br, I) [31] without explicitly considering spin–orbit coupling effects or evaluating their feasibility for laser cooling applications. In the present work, we extend this line of research to CdBr, performing high-level ab initio calculations that explicitly include spin–orbit coupling to provide accurate potential energy curves and transition properties relevant for assessing its suitability as a candidate for laser cooling studies.

Despite its chemical and environmental importance, the CdBr molecule has received limited attention in the literature, and its potential as a candidate for laser cooling remains unexplored. Experimentally, Huber and Herzberg [32] summarized a comprehensive compilation of all experimental data on the ground and low-lying excited states of the CdBr molecule. The first emission spectra of CdBr in the 307.5–324.7 nm region were examined by Wieland (1929) [33], who identified a component of the ^2^Π–^2^Σ (C–X) system with its (0,0) band at 317.7 nm and reported three additional bands attributed to another electronic system. Later, Howell [34] indicated that the probable (0,0) bands in Wieland’s measurements lie at 317.7–329.6 nm. Then, Gosav et al. [35] measured the UV absorption spectrum of CdBr produced by co-photolysis and observed both ^2^Π_3/2_-^2^Σ and ^2^Π_1/2_-^2^Σ systems. Ediger et al. [4] achieved lasing on the $B^{2}\Sigma_{1/2}^{+}\to \;X^{2}\Sigma_{1/2}^{+}$ bands of CdBr at 811 nm by using an ArF laser to photodissociate CdBr_2_; from these measurements, they determined that the radiative lifetime for the $v^{\prime }=0$ level of $B^{2}\Sigma_{1/2}^{+}$ is 25±4 ns.

On the theoretical side, early work by Liao et al. [36] provided spectroscopic constants for the X^2^Σ^+^ state of CdBr using Density Functional Theory (DFT). Subsequently, Shepler et al. [37] performed extensive computations of the ground state X^2^Σ^+^ equilibrium structures and spectroscopic constants at the coupled cluster singles and doubles with perturbative triples level (CCSD(T)) by treating the core-valence (CV) correlation effect, the spin–orbit coupling (SOC) effect, and the scalar relativistic effects. Later, in 2007, Cheng et al. [38] optimized the ground-state geometry of CdBr and reported its spectroscopic constants, dissociation energy, and dipole moment. Extending beyond the ground state, Badreddine et al. (2017) [31] performed an intensive study on the 10 low-lying Λ-S states at the MRCI+Q level and determined their spectroscopic parameters. More recently (2018), Li et al. [39] carried out a comprehensive icMRCI+Q study of 14 Λ-S states, as well as 30 Ω states, deriving their spectroscopic constants. They also examined predissociation in the $C^{2}\Pi (v^{\prime }\geq 1)\to B^{2}\Sigma^{+}$ transition using the SOC matrix elements and calculated lifetimes for $v^{\prime }=0–5$ in the $C^{2}\Pi$ and $B^{2}\Sigma^{+}$ states.

In this work, the electronic structure and transition properties of the low-lying excited states of CdBr are investigated. Ab initio CASSCF/MRCI + Q calculations are employed to compute the adiabatic potential energy curves (PECs) and the permanent dipole moment curves (PDMCs) in the ^2S+1^Λ^+/−^ and Ω^(±)^ representations, both with and without spin–orbit coupling, respectively. For the bound states, the spectroscopic parameters (T_e_, R_e_, ω_e_, B_e_, $\alpha_{e}$, $\mu_{e}$, and D_e_) are calculated and analyzed. In addition, the rovibrational constants (E_v_, B_v_, D_v_, R_min_, and R_max_) are presented for the investigated bound states with and without the spin–orbit coupling. Based on these data, the transition dipole moments, the Franck−Condon factors, the Einstein coefficient, the radiative lifetimes, the vibrational branching ratio, and the slowing distance are determined for the transition X^2^Π_1/2_ − Ξ^2^Σ^+^_1/2_. These results show that the molecule CdBr is a good candidate for laser cooling and an optical laser cooling scheme is proposed by utilizing four lasers at a wavelength in the ultraviolet region, reaching a sub-microkelvin temperature limit.

## 2. Results and Discussion

### 2.1. The Potential Energy Curves and Spectroscopic Constants

**Figure 1:**
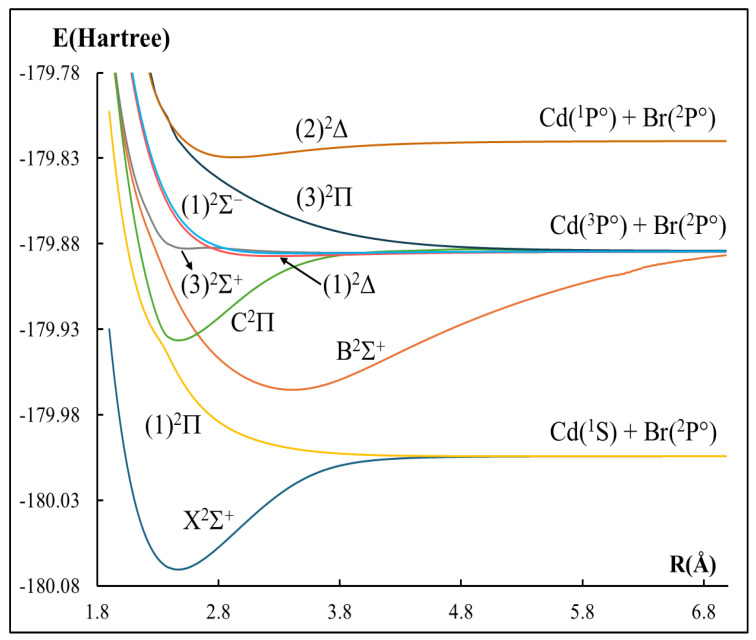
Potential energy curves of the doublet electronic states for the CdBr molecule without the spin–orbit coupling effect.

**Figure 2:**
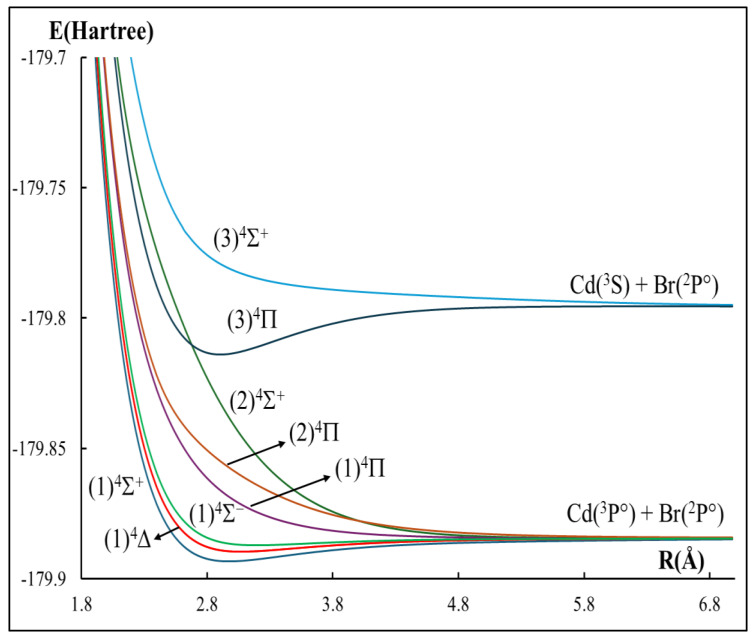
Potential energy curves of the quartet electronic states for the CdBr molecule without the spin–orbit coupling effect.

**Figure 3:**
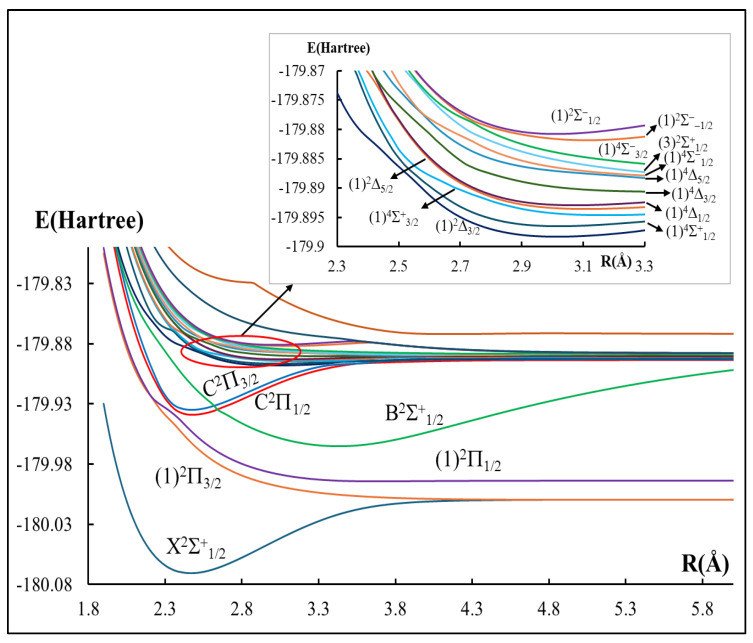
Potential energy curves of the doublet and quartet electronic states for the CdBr molecule with the spin–orbit coupling effect.

The low-lying spin-free potential energy curves of the seven doublets and eight quartets electronic states of the CdBr molecule have been calculated and presented in Figure 1 and Figure 2, while those with spin–orbit coupling are given in Figure 3. In total, 15 Λ-S states correlating with the four lowest dissociation asymptotes Cd(^1^S) + Br(^2^P°), Cd(^3^P°) + Br(^2^P°), Cd(^1^P°) + Br(^2^P°), and Cd(^3^S) + Br(^2^P°) are calculated at the MRCI+Q level of theory. At the asymptotic limit of the separated atoms, cadmium (Cd) is in a singlet ^1^S state and bromine (Br) in a doublet ^2^P° state (odd parity). Following the correlation rules summarized by Huber and Herzberg [32], the combination of these atomic levels yields the formation of the molecular states X^2^Σ^+^ and (1)^2^Π states. Figure 1 confirms that our curves reproduce these states, which constitute the first dissociation channel. The second dissociation asymptote Cd(^3^P°) + Br(^2^P°) correlates with a set of bound states B^2^Σ^+^, C^2^Π, (1)^4^Δ, (1)^4^Σ^+^, (3)^2^Σ^+^, (1)^2^Δ, (1)^4^Σ, and (1)^2^Σ^−^ and predominantly repulsive states, namely (1)^4^Π, (2)^4^Π, (3)^2^Π, and (2)^4^Σ^+^. Similarly, higher channels also follow the expected dissociation correlations where (2)^2^Δ state correlates correctly with Cd(^1^P°) + Br(^2^P°), and (3)^4^Π and (3)^4^Σ^+^ states are attributed to the Cd(^3^S) + Br(^2^P°) dissociation channel. Any additional states not shown at specific limits are absent simply because they fall outside the set of states examined in the present work. A comparison of our calculated asymptotic-limit energy separations $E_{\mathrm{theo}}$ (between each higher dissociation channel and the lowest one, without spin–orbit coupling) with the experimental atomic separations $E_{\exp}$ from the National Institute of Standards and Technology website (NIST) [40] is summarized in Table 1. The second dissociation channel lies 26,228.93 cm^−1^ above the first, whereas the experimental value is 31,246.335 cm^−1^, resulting in a relative error of 16.0%. The third channel lies at 40,452.05 cm^−1^, compared with the experimental value of 43,692.384 cm^−1^, which yields a good agreement with a percentage relative error of 7.4%. On the other hand, the fourth channel lies at 40,452.05 cm^−1^; compared with the experimental value (45,889.49 cm^−1^), this corresponds to 10.9%.

The spectroscopic parameters such as the transition energy with respect to the ground state minimum T_e_, the equilibrium bond length R_e_, the harmonic frequency ω_e_, the rotational constant B_e_, the vibration–rotation interaction constant $\alpha_{e}$, the dissociation energy D_e_, and the permanent dipole moment $\mu_{e}$ are calculated and analyzed for the investigated bound electronic states upon fitting the corresponding potential energy curve into a polynomial around the internuclear distance at equilibrium R_e_. Table 2 illustrates the spectroscopic constants for the lowest electronic states of the CdBr molecule without spin–orbit coupling. The spectroscopic constants obtained for the spin-free Λ–S states of CdBr show good overall consistency with experiment [32] and prior theory [31,36,37,38,39]. For the ground state X^2^Σ^+^, our values (R_e_ = 2.468 Å, ω_e_ = 231.4 cm^−1^, B_e_ = 5.928 cm^−1^) reproduce the available experimental data [32] very closely, with an (Obs. − Calc.) difference in only −0.9 cm^−1^ for ω_e_, confirming an accurately described equilibrium geometry and reliable representation of the potential energy surface. For the B^2^Σ^+^ state, the computed T_e_ and R_e_ values are in good agreement with earlier theoretical reports [31,39], and the corresponding vibrational constant ω_e_ follows the expected trend of bond weakening upon electronic excitation. Similarly, for the C^2^Π state, the calculated T_e_ = 29,340 cm^−1^ and ω_e_ = 268 cm^−1^ show reasonable agreement with the corresponding experimental values (31,075 cm^−1^ and 254.5 cm^−1^) [32], with deviations of 1735 cm^−1^ in $T_{e}$ (≈5.6%) and 13.5 cm^−1^ in $\omega_{e}$ (≈5.3%), which lie within the typical accuracy range of MRCI-level calculations. These results collectively confirm the internal consistency and predictive capability of the present calculations across all investigated Λ–S states of CdBr.

After considering the SOC effect, there are 18 Ω states investigated from the two lowest dissociation channels of the Λ–S states of CdBr, as shown in Figure 3. Table 3 summarizes the dissociation behavior of the lowest Ω states that arise from these dissociation asymptotes. For each channel, the energy separation from the lowest asymptote is reported along with the experimental data from the NIST Atomic Spectra Database (NIST) [40] and the corresponding percent error. Overall, the calculated energy separations in this work are in fair agreement with the experimental measurements, with an average percentage relative error of 10.5% over the examined asymptotes. As is well known, the SOC effect is very important to the spectroscopy and dynamics of molecules containing heavy atoms [41,42,43,44]. This requirement is validated by the notably large splitting energies observed for the C^2^Π state (approximately 930 cm^−1^ between C^2^Π_1/2_ and C^2^Π_3/2_) electronic states. These results highlight the significant influence of spin–orbit coupling on the electronic states of the CdBr molecule. Table 4 presents the spectroscopic constants for bound Ω states of CdBr, including spin–orbit coupling, compared to available experimental data [32] and previous theoretical calculations [31,36,37,38,39]. For the ground X^2^Σ^+^_1/2_ state, our harmonic frequency ω_e_ differs from experiment [32] by only (Obs. − Cal.) = −0.1 cm^−1^, while earlier theoretical studies [31,36,37,38,39] reported deviations ranging from 2.7 to 32 cm^−1^. The excited (1)^2^Π_1/2_ state is found at T_e_ = 1678 cm^−1^ and exhibits a small vibrational frequency ω_e_ = 40.3 cm^−1^, indicating a weak bonding. The splitting of C^2^Π state is also well reproduced: C^2^Π_1/2_ is predicted at T_e_ = 28,860 cm^−1^ with (Obs. − Cal.) = 1440 cm^−1^ (4.8%), and C^2^Π_3/2_ at T_e_ = 29,790 cm^−1^ with (Obs. − Cal.) = 1673 cm^−1^ (5.3%), preserving the correct spin–orbit ordering with the experiment. For C^2^Π_3/2_, our ω_e_ deviates from experiment by only 0.2 cm^−1^ compared with 17.6 cm^−1^ in prior calculation [39]. Overall, the explicit treatment of spin–orbit coupling yields spectroscopic parameters that follow experimental trends for the low-lying states and offers quantitatively reliable vibrational constants where benchmarks exist.

### 2.2. Rovibrational Calculations

A theoretical calculation of the rovibrational constants for different vibrational levels of a given transition can predict the absorption/emission line positions in the spectrum of a molecule [45,46]. Moreover, vibrational states of molecules have been suggested as qubit encodings for quantum computers [47]. By using the canonical function approach [48,49,50] and the cubic spline interpolation of the PEC between every two consecutive points, the rovibrational constants such as the vibrational energy E_ν_, the rotational constant B_ν_, the centrifugal distortion constant D_ν_, and the abscissas of turning points R_min_ and R_max_ have been calculated for the lowest vibrational levels of the CdBr molecule for states in both the (Λ-S) and Ω representations. The rovibrational constants of the states X^2^Σ_1/2_ and C^2^Π_1/2_ are presented in Table 5. The values for five electronic states are provided up to *v* = 75 for the spin-free and two electronic states up to *v* = 14 for the spin–orbital coupling, as listed, respectively, in Tables S1 and S2 of the Supplementary Materials. To the best of current knowledge, no prior rovibrational constants for CdBr are available for direct comparison. The present values, therefore, constitute the first comprehensive dataset for this molecule.

### 2.3. Transition Properties and Laser Cooling Scheme

Laser cooling is a process that slows atoms or molecules by repeatedly scattering photons through fast and well-controlled optical transitions [51]. The momentum transfer from each scatter decreases the sample’s kinetic energy and entropy. Although molecules can also be cooled and trapped by direct methods [52,53,54,55] (e.g., buffer gas cooling, Stark deceleration) or by indirect routes [56,57] (e.g., photoassociation). Laser cooling has achieved sub-millikelvin temperatures across many diatomic [58,59,60,61] and linear triatomic systems [62,63,64]. Selecting appropriate cooling transitions among vibronic levels in a diatomic molecule requires the consideration of several important criteria, which are detailed below.

#### 2.3.1. Highly Diagonal Franck–Condon Array

A highly diagonal Franck–Condon pattern in the considered band system minimizes the number of repump lasers and maintains a closed cycling loop; this condition is typically identified by a very small difference in the equilibrium internuclear separation (ΔR_e_) between the involved electronic states [65,66].

#### 2.3.2. Absence of Intervening Electronic States

An intervening state is an intermediate level between the excited and ground states of the loop that has a sufficiently large transition probability from the excited state to act as a loss channel; if that probability is small, the state is non-intervening. Recent studies indicate that intermediate states can still be incorporated into the cooling dynamics in some schemes [67].

#### 2.3.3. Short Radiative Lifetimes

The radiative lifetime of the chosen vibrational transition should be short to achieve high photon-scattering rates during the cooling process; typical useful lifetimes fall in the ns–ms range [68,69].

**Figure 4:**
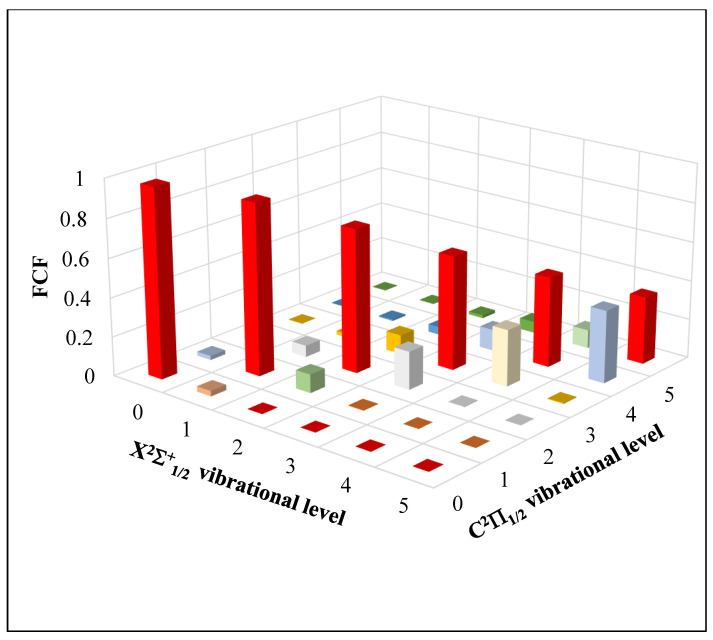
Franck–Condon factor (FCF) for the transition between the X^2^Σ^+^_1/2_ and C^2^Π_1/2_ electronic states of the CdBr molecule.

Through the comparison of the values of the internuclear distance R_e_ of the two electronic states X^2^Σ^+^_1/2_ and C^2^Π_1/2_ in Table 4, a shows a very small difference, ΔR_e_ = 0.007 Å, which leads to a diagonal Franck–Condon factor (FCF) for the transition between vibrational levels of these two electronic states. The calculated values of the Franck–Condon factor (FCF) for the transition C^2^Π_1/2_ − X^2^Σ^+^_1/2_ are represented in Figure 4. With the diagonality of the FCF for this transition, the first criterion (Section 2.3.1) of a closed laser cooling cycle is verified.

As shown in Figure 3, there are two unbound intervening states (1)^2^Π_1/2_ and (1)^2^Π_3/2_ between the two considered electronic states X^2^Σ^+^_1/2_ and C^2^Π_1/2_, which have no influence on the laser cycling loop between these two because of the very low transition probability between C^2^Π_1/2_-(1)^2^Π_3/2_ and C^2^Π_1/2_-(1)^2^Π_1/2_ [70]. Consequently, these states do not perturb the laser cooling cycle between the X^2^Σ^+^_1/2_ and C^2^Π_1/2_ states. Hence, the second criterion (Section 2.3.2.), the absence of intervening states, is fulfilled.

**Figure 5:**
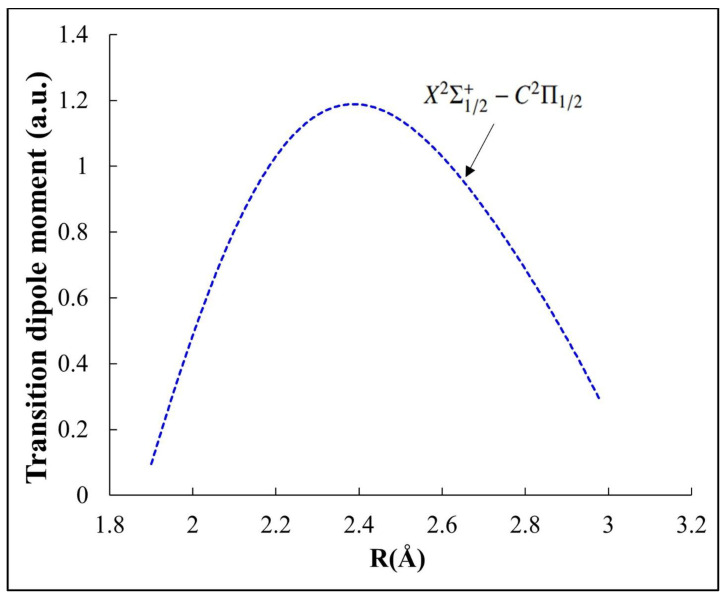
Transition dipole moment curve of the X^2^Σ^+^_1/2_ − C^2^Π_1/2_ transition of the CdBr molecule.

Aside from the diagonality of the FCF and the absence of intervening states, a sufficiently short lifetime (criterion (iii)) is desirable to ensure a sufficient photon scattering rate for rapid laser cooling. This radiative lifetime (τ_v′_) can be evaluated by the inverse of the Einstein coefficient A_v′v_, (τ_v′_ = 1/Σ_v_A_v′v_). The vibrational Einstein Coefficient among the transition X^2^Σ^+^_1/2_ − C^2^Π_1/2_ is calculated using the LEVEL 11 program [71] according to the following formula [46]:

(1) $$A_{v\prime v}=\frac{(3.1361891)(10^{-7})(∆E{)}^{3}(\left\langle \psi_{\nu \prime }\right|M(r)\left|\psi_{\nu }\right\rangle {)}^{2}}{2}$$

where A_νν′_ has as units s^−1^, ∆E is the emission frequency (in cm^−1^) and M(r) is the electronic transition dipole moment between the two considered electronic states X^2^S^+^_1/2_ and C^2^P_1/2_ calculated by using the MOLPRO program [72] and represented in Figure 5.

The vibrational branching ratio (*R*_*ν*′*ν*_), which represents the transition probability between two vibrational levels of the considered electronic states, is obtained by using the following formula [73]:

(2) $$R_{\nu \prime \nu }\;=\;\frac{A\nu \prime \nu }{\sum A\nu \prime \nu }$$

**Figure 6:**
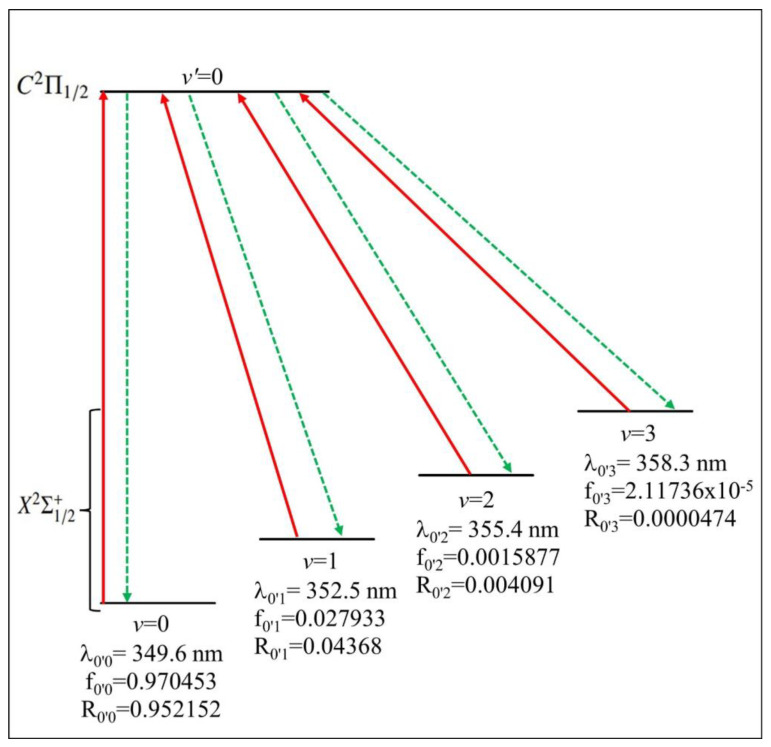
Laser cooling scheme of the X^2^Σ^+^_1/2_ − C^2^Π_1/2_ transition of the CdBr molecule.

The corresponding calculated values of Einstein coefficients and the vibrational branching ratio are given in Table 6, along with the values of the radiative lifetime. However, with the diagonal FCF (Figure 4), the short radiative lifetime (of 16.07 ns < t < 41.35 ns) for the transition C^2^Π_1/2_ − X^2^Σ^+^_1/2_ and the absence of an influencing intervening electronic state, the CdBr molecule is a good candidate for Doppler laser cooling. For this transition, our suggested laser cooling scheme is given in Figure 6. In the ultraviolet range, the red lines represent the cycling pumping lasers, where the wavelength of the main pumping laser is λ_0′0_= 349.6 nm. To close the leaks from the higher vibrational level *v*′ = 0 of the electronic state, C^2^Π_1/2_, there is a need for the three repumping lasers of wavelengths λ_0′1_ = 352.5 nm, λ_0′2_ = 355.4 nm, and λ_0′3_ = 358.3 nm, where the dotted green lines represent the spontaneous decay from the higher electronic state. For the transition C^2^Π_1/2_ − X^2^Σ^+^_1/2_ of the CdBr molecule. In Figure 6, f_0′v_ (*v* = 0, 1, 2, 3) denotes the calculated Franck–Condon factors for spontaneous decay from the excited level *v*′ = 0 to the ground-state level *v*, and R_0′*v*_ (*v* = 0, 1, 2, 3) represent the vibrational branching ratios, which correspond to the transition probabilities for spontaneous emission from the *v*′ = 0 of C^2^Π_1/2_ to *v* of X^2^Σ^+^_1/2_. For this transition, the total number N of cycles for photon absorption/emission, which is the reciprocal of the total loss, is given by

N = 1/(1 − ɳ) = 34,573(3)

with ɳ _=_ R_0′0_ + R_0′1_ + R_0′2_ + R_0′3_ [74]. The corresponding experimental parameters for the considered transitions are as follows [75]:

(4) $$V_{rms}=\frac{hN}{m\lambda_{00}}\;=\;207.2\;m/s$$

(5) $$T_{\mathrm{ini}}=\frac{mV^{2}\;}{2k_{B}}\;=\;496.0\;K$$

(6) $$a_{\max}=\frac{hN_{e}}{N_{\text{tot}}m\lambda_{00}\tau }\;=\;3.89\times 10^{4}\;m/s^{2}$$

(7) $$L_{\text{min}}=\frac{k_{B}T_{\text{ini}}}{ma_{\text{max}}}\;=\;55.2\;\mathrm{cm}$$

T_D_ = 1.24 × 10^−4^ K and T_r_ = 8.31 10^−7^ K (8)

where h, m, k_B_, T_ini,_ and a_max_ represent the Planck constant, the mass, the Boltzmann constant, the initial temperature, and the maximum acceleration of the molecule, respectively; V_rms_ is the rms velocity, L_min_ is the minimum slowing distance. In the main cycling transition, N_e_ is the number of excited states, and N_tot_ is the number of excited states connected to the ground state plus N_e_. T_r_ and T_D_ are the recoil and the Doppler temperatures, respectively.

One must be cautious, however, that the proposed parameters in this work are primarily based on theoretical work. Consequently, one should pay attention to various caveats before proceeding with any experimental initiative. We cite a few in the following:

(1) Our calculations show a T_e_ value for the state C^2^Π_1/2_ that differs by about 1400 cm^−1^ with respect to previous experimental work. Even though minimal when taking into account the state C^2^Π_1/2_ is a high-lying state, ~30,000 cm^−1^ above the ground state, such a difference has still to be considered when setting up pumping laser wavelengths.
(2) The calculated harmonic vibrational frequency ω_e_, shows very good agreement with the experiment for the ground X^2^Σ^+^_1/2_ and C^2^Π_3/2_ states. An experimental validation of the same parameter for the C^2^Π_1/2_ state is highly recommended.
(3) A preliminary investigation of the position of the vibrational level v′ = 0 has shown no crossings with the neighboring C^2^Π_3/2_ and B^2^Σ^+^_1/2_; however, a direct confirmation is yet to be made available.

## 3. Materials and Methods

The present theoretical work is based on ab initio calculations, which are performed using the state-averaged complete active space self-consistent field (CASSCF) method followed by the multi-reference configuration interaction (MRCI) method with Davidson correction (+Q) [76]. All computations are carried out using the computational chemistry program MOLPRO 2015.1 package [72], taking advantage of the graphical interface program GABEDIT [77].

The Cadmium (Cd) atom, consisting of forty-eight electrons, has been modeled using the relativistic energy-consistent pseudopotential ECP28MDF [78] together with the aug-cc-pV5Z-PP basis set [78,79], including *spdfg* functions. In this pseudopotential, twenty-eight electrons are frozen in the core of Cd, and the remaining twenty electrons are left for explicit treatment. For bromine (Br), the relativistic ECP28MDF pseudopotential has been used in combination with its (6s6p)/[4s4p] valence basis [80], replacing twenty-eight core electrons, and treating the remaining seven electrons explicitly. A complete active space self-consistent field (CASSCF) calculation was performed with seven valence electrons from CdBr distributed over nine molecular orbitals; hence, this active space is referred to as CAS(7,9). The active symmetry molecular orbitals 5a_1_, 2b_1_, 2b_2_, and 0a_2_ are distributed into the irreducible representation of the C_2v_ point group symmetry. These arise from five σ-type orbitals (Cd: 5s, 5p_0_, 6s, Br: 4p_0_, 5s) and two π-type orbital pairs (Cd: 5p_±1_, Br: 4p_±1_). In the subsequent MRCI+Q calculations, the Cd (4s^2^ 4p^6^ 4d^10^) and Br (4s^2^) orbitals were kept as closed-shell orbitals and included in the correlation treatment.

## 4. Conclusions

A comprehensive ab initio study of the CdBr molecule was performed to investigate its electronic structure and assess its potential for direct laser cooling. Calculations based on a complete active space self-consistent field (CASSCF)/(MRCI + Q) method yielded 15 spin-free electronic states and 18 spin–orbit electronic states. The adiabatic potential energy curves for the ground and excited states, together with derived spectroscopic (T_e_, R_e_, ω_e_, B_e_, $\alpha_{e}$, $\mu_{e}$, and D_e_) and rovibrational (E_v_, B_v_, D_v_, R_min_, and R_max_) constants of the bound states have been studied. The results show good agreement with previously reported data, confirming the reliability and accuracy of the computational approach. A detailed analysis of the transition dipole moment, Franck–Condon factors (FCFs), the Einstein coefficients ($A_{vv\prime }$), and the spontaneous radiative lifetimes for the transition X^2^Σ_1/2_ − C^2^Π_1/2_ reveal a strongly diagonal Franck–Condon array, the absence of perturbing intermediate electronic states, and short radiative lifetimes. These characteristics fulfill the principal criteria required for direct laser cooling of molecules. Furthermore, vibrational branching ratios, the number of photon absorption–emission cycles (N), and the recoil and Doppler temperature limits were estimated, providing essential parameters for experimental implementation. A feasible laser cooling scheme is proposed, employing four pumping and repumping lasers in the ultraviolet region. Overall, the present results highlight CdBr as a promising candidate for direct laser cooling, opening up opportunities for future experimental investigations.

## Figures and Tables

**Table 1:** The dissociation limits of the Λ-S states of the CdBr molecule.

Atomic States Cd + Br	Molecular States CdBr	Energy Separation (cm^−1^)		
		E_theo_. ^a^	E_exp_. ^b^	% Relative Error
Cd (4d^10^5s^2^, ^1^S) + Br (4s^2^4p^5^, ^2^P°)	X^2^Σ^+^, (1)^2^Π	0.00	0.00	0.00
Cd (4d^10^5s5p, ^3^P°) + Br (4s^2^4p^5^, ^2^P°)	B^2^Σ^+^, X^2^Π, (1)^4^Δ, (1)^4^Σ^+^, (3)^2^Σ^+^, (1)^2^Δ, (1)^4^Σ^−^, (1)^4^Π, (2)^4^Π, (2)^4^Σ^+^ (3)^2^Π, (1)^2^Σ^−^	26,245.15	31,246.335	16.0
Cd (4d^10^5s5p, ^1^P°) + Br (4s^2^4p^5^, ^2^P°)	(2)^2^Δ	40,452.05	43,692.384	7.4
Cd (4d^10^5s6s, ^3^S) + Br (4s^2^4p^5^, ^2^P°)	(3)^4^Π, (3)^4^Σ^+^	45,889.49	51,483.980	10.9

^a^ Present work.; ^b^ J-weighted averaged values, from ref. [40]. Note that the table is energy ordered.

**Table 2:** Spectroscopic constants of bound Λ-S states (spin-free) of the CdBr molecule. Numbers in parentheses, given in italics, represent (Obs. − Calc.) values based on the only available experimental data [32], shown in bold.

State	Ref.	T_e_ (cm^−1^)	R_e_ (Å)	ω_e_ (cm^−1^)	B_e_ (cm^−1^)	$\alpha_{e}$ (cm^−1^)	D_e_ (cm^−1^)	µ_e_ (a.u)
X^2^Σ^+^	**Exp. [32]**	**0**	-	**230.5**	-	-	**7258–12,904**	
	This work	0	2.468	231.4 *(−0.9)*	5.928	0.2483	14,552	1.196
	Calc. [36]	0	2.52	214 *(16.5)*	-	-	-	-
	Calc. [37]	0	2.4664	233.2 *(−2.7)*	-	-	11,856	-
	Calc. [38]	0	2.563	198 *(32.5)*	-	-	11,130	1.395
	Calc. [31]	0	2.564	210.5 *(20)*	5.48	-		-
	Calc. [39] *	0	2.4738	227.7 *(2.8)*	5.91	-	12,098	-
	Calc. [39] **	0	2.5206	223.3 *(7.2)*	5.69	-	14,840	-
B^2^Σ^+^	This work	23,033	3.410	118.4	3.104	-	17,771	1.555
	Calc. [31]	19,528	3.4679	119.0	3.00	-		-
	Calc. [39] *	22,272	3.2646	133.9	3.39	-	19,841	-
	Calc. [39] **	21,573	3.5386	113.2	2.89	-	20,405	-
C^2^Π	**Exp. [32]**	**31,075**	-	**254.5**	-	-	-	-
	This work	29,340 *(1735)*	2.466	268.0 *(−13.5)*	5.939	0.7678	11,441	1.013
	Calc. [31]	28,850 *(2225)*	2.5522	222.1 *(32.4)*	5.53	-	-	-
	Calc. [39] *	30,688 *(387)*	2.4462	243.2 *(11.3)*	6.04	-	11,533	-
	Calc. [39] **	30,333 *(742)*	2.4973	259.4 *(−4.9)*	5.80	-	11,937	-
(1)^4^Σ^+^	This work	38,853	2.975	85.1	4.078	0.5183	1933	0.860
	Calc. [31]	35,937	3.3823	46.9	3.13	-	--	-
	Calc. [39] *	40,395	3.0194	77.2	3.97	-	1774	-
(1)^4^Δ	This work	39,683	3.071	68.9	3.827	0.5121	1123	0.829
	Calc. [31]	36,491	3.6213	32.3	2.75	-	-	-
	Calc. [39] *	41,035	3.1112	64.5	3.74	-	1209	-
(1)^2^Δ	This work	40,220	3.267	49.3	3.349	1.8016	587	0.624
	Calc. [31]	36,647	4.0051	24.8	2.26	-	-	-
	Calc. [39] *	41,475	3.3015	45.8	3.32	-	725	-
(1)^4^Σ^−^	This work	40,243	3.194	51.0	3.538	0.8123	568	0.733
	Calc. [39] *	41,504	3.2263	51.5	3.48	-	725	-
(1)^2^Σ^−^	This work	40,548	3.428	29.9	3.069	0.9923	263	0.536
	Calc. [31]	36,803	4.4471	-	1.68	-	-	-
	Calc. [39] *	41,730	3.3858	38.6	3.15	-	483	-
(3)^4^Π	This work	56,280	2.904	128.6	4.278	0.2765	4050	0.784
	Calc. [31]	54,565	3.0514	99.99	3.88	-	-	1.7874

* Evaluated values using MRCI+Q, including the correlated effect of 4d^10^ electrons of Cd; ** Evaluated values using MRCI+Q with exclusion of correlated effect of 4d^10^ electrons of Cd.

**Table 3:** The lowest dissociation limits of possible Ω states of the CdBr molecule obtained by MRCI+Q calculations in comparison with the experimental values.

Atomic State	Possible Ω States	Relative Energy (cm^−1^)		
		This Work	Exp. [40]	% Relative Error
Cd (^1^S) + Br (^2^P_3/2_)	1/2, 3/2	0.00	0.00	0.00
Cd (^1^S) + Br (^2^P_1/2_)	1/2	3580.42	3685.24	2.8
Cd (^3^P_0_) + Br (^2^P_3/2_)	1/2, 3/2	25,634.94	30,113.990	14.9
Cd (^3^P_1_) + Br (^2^P_3/2_)	5/2, 3/2, 3/2, 1/2, 1/2, 1/2	26,586.12	30,656.087	13.8

**Table 4:** Spectroscopic constants of bound W states (spin–orbit coupling) of the CdBr. Numbers in parentheses, given in italics, represent (Obs. − Calc.) values based on the only available experimental data [32], shown in bold.

State	Ref.	T_e_ (cm^−1^)	R_e_ (Å)	ω_e_ (cm^−1^)	B_e_ × 10^2^ (cm^−1^)
X^2^Σ^+^_1/2_	**Exp. [32]**		-	**230.5**	-
	This work	0.0	2.468	230.6 *(−0.1)*	5.92
	Calc. [36]		2.52	214.0 *(16.5)*	-
	Calc. [37]		2.4664	233.2 *(−2.7)*	-
	Calc. [38]		2.563	198 *(32.5)*	-
	Calc. [31]		2.564	210.5 *(20)*	5.48
	Calc. [39]		2.4749	226.8 *(3.7)*	-
(1)^2^Π_1/2_	This work	16,798	3.681	40.3	2.66
B^2^Σ^+^_1/2_	This work	23,143	3.431	118.1	3.07
	Calc. [39]	22,263	3.2798	133.3	3.36
C^2^Π_1/2_	**Exp. [32]**	**30,300**	-	-	-
	This work	28,860 *(1440)*	2.475	271.1	5.89
	Calc. [39]	30,140 *(160)*	2.4582	199.8	5.64
C^2^Π_3/2_	**Exp. [32]**	**31,463**	-	**253.8**	-
	This work	29,790 *(1673)*	2.469	254.0 *(−0.2)*	5.92
	Calc. [39]	31,148 *(315)*	2.447	236.2 *(17.6)*	6.05
(1)^4^Σ^+^_1/2_	This work	38,212	3.022	4.03	3.954
(1)^4^Σ^+^_3/2_	This work	38,628	3.170	1.40	3.619
(1)^4^Δ_1/2_	This work	39,001	3.072	9.00	3.825

**Table 5:** The rotational constants for the ground X^2^Σ_1/2_ and C^2^Π_1/2_ states with spin–orbit of the CdBr.

X^2^Σ_1/2_					
*v*	E*_v_* (cm^−1^)	B*_v_* × 10^2^ (cm^−1^)	D*_v_* × 10^8^ (cm^−1^)	R_min_ (Å)	R_max_ (Å)
0	115.19	5.8462	1.4505	2.417	2.526
1	349.73	5.8373	1.5152	2.375	2.569
2	581.02	5.8211	1.3922	2.353	2.601
3	813.33	5.7900	1.1881	2.336	2.627
4	1049.29	5.7586	1.4168	2.320	2.652
5	1283.51	5.7470	1.6042	2.307	2.574
6	1513.97	5.7277	1.2946	2.295	2.695
7	1744.61	5.6985	1.3159	2.284	2.714
8	1974.77	5.6779	1.5293	2.274	2.733
9	2202.70	5.6614	1.4943	2.265	2.751
10	2428.84	5.6381	1.2984	2.256	2.769
11	2654.49	5.6127	1.4365	2.248	2.786
12	2878.74	5.5948	1.5606	2.240	2.802
13	3100.99	5.5740	1.3751	2.233	2.819
14	3322.25	5.5495	1.4202	2.226	2.835
15	3542.24	5.5290	1.5158	2.220	2.850
16	3760.60	5.5079	1.4508	2.213	2.866
17	3977.64	5.4854	1.4603	2.207	2.881
18	4193.31	5.4637	1.4804	2.202	2.896
19	4407.55	5.4416	1.4832	2.196	2.911
20	4620.39	5.4198	1.5030	2.191	2.926
C^2^Π_1/2_					
*v*	E*_v_* (cm^−1^)	B*_v_* × 10^2^ (cm^−1^)	D*_v_* × 10^8^ (cm^−1^)	R_min_ (Å)	R_max_ (Å)
0	133.93	5.8522	1.0877	2.430	2.534
1	403.34	5.8033	1.0410	2.401	2.580
2	672.20	5.7602	0.92432	2.381	2.615
3	944.52	5.7295	0.77253	2.368	2.639
4	1223.15	5.6966	0.85974	2.358	2.663
5	1500.68	5.6366	1.1576	2.348	2.689
6	1767.48	5.5764	1.0529	2.340	2.714
7	2029.13	5.5422	1.0365	2.333	2.737
8	2287.83	5.5088	1.0548	2.326	2.758
9	2543.54	5.4768	1.1232	2.319	2.779
10	2795.01	5.4347	0.99751	2.313	2.799
11	3044.11	5.4035	1.0633	2.308	2.819
12	3290.20	5.3635	1.1783	2.302	2.838
13	3532.47	5.3357	1.1823	2.297	2.857

**Table 6:** The radiative lifetimes τ and the vibrational branching ratio of the vibrational transitions between the electronic states X^2^Σ^+^_1/2_ − C^2^Π _1/2_ of the CdBr molecule.

CdBr X^2^Σ^+^_1/2_ − C^2^Π_1/2_								
	ν′(C^2^Π_1/2_) = 0		1	2	3	4	5	6
ν (X^2^Σ^+^_1/2_) = 0	A_ν′*v*_	30,882,256.77	1,746,179.3	385,206.7187	127,778.62	42,465.012	13,967.007	4883.3049
	R_ν′*v*_	0.95215209	0.02805663	0.00630063	0.00212631	0.00072613	0.00024938	0.00020192
ν = 1	A_ν′*v*_	1,416,739.24	55,179,978	4,685,587.143	1,408,663.4	551,980.73	209,044.62	76,279.25
	R_ν′*v*_	0.04368046	0.88660102	0.07663978	0.02344095	0.00943862	0.00373242	0.00315406
ν = 2	A_ν′*v*_	132,692.0758	5,016,532.9	45,183,875.21	7,673,534	2,848,681.2	1,181,868.7	537,702.81
	R_ν′*v*_	0.00409112	0.08060284	0.73904983	0.12769189	0.04871113	0.02110184	0.02223341
ν = 3	A_ν′*v*_	1537.496581	289,325.54	10,456,180.64	34,819,514	8,703,381.1	3,984,275.9	1,833,160.8
	R_ν′*v*_	0.00004740	0.00464872	0.17102647	0.57941614	0.14882380	0.07113780	0.07579917
ν = 4	A_ν′*v*_	429.5835471	19.191812	397,518.6415	15,396,833	26,303,405	7,904,342.2	4,730,441.4
	R_ν′*v*_	0.00001324	0.00000031	0.00650201	0.25621189	0.44977608	0.14112917	0.19559851
ν = 5	A_ν′*v*_	469.0557706	2659.4756	22,832.35746	519,034.52	19,480,616	18,272,568	7,084,431
	R_ν′*v*_	0.00001446	0.00004273	0.00037346	0.00863702	0.33310954	0.32625008	0.29293337
ν = 6	A_ν′*v*_	39.50936826	2971.6815	6592.117738	148,781.89	550,582.32	24,441,791	9,917,546.6
	R_ν′*v*_	0.00000122	0.00004775	0.00010782	0.00247581	0.00941470	0.43639932	0.41007956
Sum(s^−1^) = A_ν′*v*_		32,434,163.73	62,237,666	61,137,792.82	60,094,139	58,481,112	56,007,857	24,184,445
τ:(s) = /A_ν′*v*_		3.08317 × 10^−8^	1.607 × 10^−8^	1.63565 × 10^−8^	1.664 × 10^−8^	1.71 × 10^−8^	1.785 × 10^−8^	4.135 × 10^−8^
τ:(ns)		30.83	16.07	16.36	16.64	17.10	17.85	41.35

## Data Availability

The original contributions presented in this study are included in the article/Supplementary Materials. Further inquiries can be directed to the corresponding author.

## Supplementary Materials

The following supporting information can be downloaded at: https://www.mdpi.com/article/10.3390/ijms27010184/s1.
