# Supplementary material for: Fine Structure Investigation and Laser Cooling Study of the CdBr Molecule
Source: Int J Mol Sci. 2025 Dec 23;27(1):184. doi: 10.3390/ijms27010184 (PMC12785689; doi:10.3390/ijms27010184)
Supplement: Supplementary file 1 [file ijms-27-00184-s001.zip › ijms-3940783-supplementary.pdf]

**Table S1.** The rovibrational constants for the different vibrational levels of different electronic states of the spin-free CdBr molecule.

| $(2)^2\Sigma^+$ | | | | | |
|---|---|---|---|---|---|
| v | $E_v$ ($cm^{-1}$) | $B_v\times10^{-2}$ ($cm^{-1}$) | $D_v\times10^{-9}$ ($cm^{-1}$) | $R_{min}$ (Å) | $R_{max}$ (Å) |
| 0 | 58.82 | 3.0704 | 8.4027 | 3.332 | 3.488 |
| 1 | 293.31 | 3.0726 | 8.6200 | 3.238 | 3.587 |
| 2 | 410.06 | 3.0734 | 8.7227 | 3.207 | 3.621 |
| 3 | 526.48 | 3.0740 | 8.8249 | 3.181 | 3.651 |
| 4 | 642.58 | 3.0743 | 8.9281 | 3.158 | 3.677 |
| 5 | 758.34 | 3.0744 | 9.0259 | 3.136 | 3.702 |
| 6 | 873.76 | 3.0743 | 9.1255 | 3.117 | 3.725 |
| 7 | 988.85 | 3.0740 | 9.2247 | 3.098 | 3.747 |
| 8 | 1103.59 | 3.0735 | 9.3192 | 3.081 | 3.768 |
| 9 | 1217.99 | 3.0729 | 9.4146 | 3.065 | 3.788 |
| 10 | 1332.05 | 3.0721 | 9.5092 | 3.050 | 3.807 |
| 11 | 1445.75 | 3.0711 | 9.6056 | 3.035 | 3.826 |
| 12 | 1559.10 | 3.0699 | 9.6952 | 3.021 | 3.844 |
| 13 | 1672.09 | 3.0686 | 9.7948 | 3.008 | 3.862 |
| 14 | 1784.73 | 3.0670 | 9.8702 | 2.996 | 3.879 |
| 15 | 1897.01 | 3.0654 | 9.9593 | 2.983 | 3.896 |
| 16 | 2008.93 | 3.0636 | 10.030 | 2.971 | 3.913 |
| 17 | 2231.69 | 3.0595 | 10.186 | 2.949 | 3.945 |
| 18 | 2342.54 | 3.0572 | 10.270 | 2.938 | 3.960 |
| 19 | 2453.02 | 3.0548 | 10.345 | 2.928 | 3.977 |
| 20 | 2563.14 | 3.0522 | 10.425 | 2.917 | 3.992 |
| 21 | 2672.90 | 3.0496 | 10.483 | 2.908 | 4.007 |
| 22 | 2782.31 | 3.0468 | 10.538 | 2.898 | 4.022 |
| 23 | 2891.35 | 3.0439 | 10.586 | 2.889 | 4.037 |
| 24 | 3000.05 | 3.0409 | 10.622 | 2.880 | 4.052 |
| 25 | 3108.40 | 3.0379 | 10.679 | 2.871 | 4.067 |
| 26 | 3216.41 | 3.0348 | 10.739 | 2.862 | 4.081 |
| 27 | 3324.07 | 3.0314 | 10.931 | 2.854 | 4.096 |
| 28 | 3431.36 | 3.0274 | 11.178 | 2.845 | 4.110 |
| 29 | 3538.21 | 3.0230 | 11.353 | 2.837 | 4.125 |
| 30 | 3644.61 | 3.0185 | 11.196 | 2.829 | 4.139 |
| 31 | 3750.61 | 3.0148 | 10.762 | 2.822 | 4.153 |
| 32 | 3856.33 | 3.0119 | 10.620 | 2.814 | 4.167 |
| 33 | 3961.81 | 3.0086 | 11.163 | 2.807 | 4.181 |
| 34 | 4066.93 | 3.0036 | 11.868 | 2.799 | 4.195 |
| 35 | 4171.55 | 2.9981 | 11.579 | 2.792 | 4.209 |
| 36 | 4275.76 | 2.9941 | 10.747 | 2.785 | 4.223 |
| 37 | 4379.72 | 2.9909 | 11.052 | 2.779 | 4.236 |
| 38 | 4483.38 | 2.9859 | 12.067 | 2.772 | 4.250 |
| 39 | 4586.56 | 2.9800 | 11.754 | 2.765 | 4.264 |
| 40 | 4689.33 | 2.9758 | 10.825 | 2.759 | 4.278 |
| 41 | 4791.85 | 2.9720 | 11.579 | 2.752 | 4.291 |
| 42 | 4894.01 | 2.9660 | 12.279 | 2.746 | 4.305 |
| 43 | 4995.70 | 2.9606 | 11.264 | 2.740 | 4.318 |

| | | | | | |
|---|---|---|---|---|---|
| 44 | 5097.08 | 2.9567 | 11.329 | 2.734 | 4.332 |
| 45 | 5198.15 | 2.9511 | 12.329 | 2.728 | 4.346 |
| 46 | 5298.76 | 2.9451 | 11.612 | 2.722 | 4.359 |
| 47 | 5399.03 | 2.9407 | 11.401 | 2.717 | 4.373 |
| 48 | 5498.99 | 2.9351 | 12.331 | 2.712 | 4.386 |
| 49 | 5598.52 | 2.9290 | 11.728 | 2.705 | 4.400 |
| 50 | 5697.71 | 2.9243 | 11.617 | 2.700 | 4.413 |
| 51 | 5796.56 | 2.9184 | 12.349 | 2.695 | 4.426 |
| 52 | 5894.99 | 2.9124 | 11.731 | 2.690 | 4.440 |
| 53 | 5993.09 | 2.9072 | 11.919 | 2.684 | 4.453 |
| 54 | 6090.84 | 2.9010 | 12.281 | 2.679 | 4.467 |
| 55 | 6188.19 | 2.8952 | 11.763 | 2.674 | 4.480 |
| 56 | 6285.20 | 2.8896 | 12.215 | 2.667 | 4.494 |
| 57 | 6381.84 | 2.8832 | 12.120 | 2.664 | 4.507 |
| 58 | 6574.03 | 2.8713 | 12.318 | 2.654 | 4.534 |
| 59 | 6669.57 | 2.8652 | 12.023 | 2.650 | 4.547 |
| 60 | 6764.76 | 2.8591 | 12.278 | 2.645 | 4.561 |
| 61 | 6859.58 | 2.8527 | 12.199 | 2.640 | 4.574 |
| 62 | 6954.04 | 2.8466 | 12.194 | 2.636 | 4.588 |
| 63 | 7048.14 | 2.8402 | 12.333 | 2.631 | 4.601 |
| 64 | 7141.87 | 2.8339 | 12.187 | 2.627 | 4.615 |
| 65 | 7235.25 | 2.8274 | 12.382 | 2.623 | 4.628 |
| 66 | 7328.27 | 2.8210 | 12.240 | 2.618 | 4.642 |
| 67 | 7420.92 | 2.8145 | 12.365 | 2.614 | 4.655 |
| 68 | 7513.22 | 2.8079 | 12.341 | 2.610 | 4.669 |
| 69 | 7605.16 | 2.8014 | 12.358 | 2.606 | 4.682 |
| 70 | 7696.73 | 2.7947 | 12.394 | 2.602 | 4.696 |
| 71 | 7787.95 | 2.7882 | 12.370 | 2.598 | 4.709 |
| 72 | 7878.81 | 2.7814 | 12.423 | 2.594 | 4.723 |
| 73 | 7969.31 | 2.7747 | 12.400 | 2.590 | 4.736 |
| 74 | 8059.46 | 2.7680 | 12.466 | 2.586 | 4.750 |
| 75 | 8149.25 | 2.7612 | 12.413 | 2.582 | 4.763 |
| **$(2)^2\Pi$** | | | | | |
| **v** | **$E_v$ ($cm^{-1}$)** | **$B_v\times10^{-2}$ ($cm^{-1}$)** | **$D_v\times10^{-8}$ ($cm^{-1}$)** | **$R_{min}$ (Å)** | **$R_{max}$ (Å)** |
| 0 | 132.72 | 5.8230 | 1.1371 | 2.422 | 2.527 |
| 1 | 394.93 | 5.7862 | 1.1219 | 2.389 | 2.572 |
| 2 | 655.36 | 5.7487 | 1.0491 | 2.370 | 2.605 |
| 3 | 915.85 | 5.7044 | 0.9149 | 2.357 | 2.633 |
| 4 | 1178.25 | 5.6542 | 0.8382 | 2.347 | 2.659 |
| 5 | 1442.30 | 5.6065 | 0.9168 | 2.337 | 2.683 |
| 6 | 1705.24 | 5.5687 | 1.0672 | 2.330 | 2.706 |
| 7 | 1964.51 | 5.5372 | 1.0882 | 2.322 | 2.727 |
| 8 | 2220.24 | 5.5023 | 0.9788 | 2.315 | 2.748 |
| 9 | 2473.89 | 5.4635 | 0.9604 | 2.308 | 2.768 |
| 10 | 2725.52 | 5.4277 | 1.0613 | 2.302 | 2.788 |
| 11 | 2974.23 | 5.3961 | 1.1362 | 2.296 | 2.807 |
| 12 | 3219.62 | 5.3653 | 1.1425 | 2.291 | 2.826 |
| 13 | 3461.87 | 5.3339 | 1.1361 | 2.285 | 2.844 |
| 14 | 3701.17 | 5.3023 | 1.1458 | 2.280 | 2.862 |

| | | | | | |
|---|---|---|---|---|---|
| 15 | 3937.56 | 5.2707 | 1.1732 | 2.276 | 2.880 |
| 16 | 4171.02 | 5.2400 | 1.2264 | 2.271 | 2.898 |
| **$(1)^4\Sigma^+$** | | | | | |
| **v** | **$E_v$ ($cm^{-1}$)** | **$B_v \times 10^{-2}$ ($cm^{-1}$)** | **$D_v \times 10^{-9}$ ($cm^{-1}$)** | **$R_{min}$ (Å)** | **$R_{max}$ (Å)** |
| 0 | 41.95 | 3.9668 | 3.6249 | 2.890 | 3.075 |
| 1 | 124.14 | 3.9168 | 3.7641 | 2.836 | 3.160 |
| 2 | 203.97 | 3.8661 | 3.9153 | 2.802 | 3.225 |
| 3 | 281.42 | 3.8146 | 4.0589 | 2.777 | 3.284 |
| 4 | 356.56 | 3.7624 | 4.2113 | 2.756 | 3.339 |
| 5 | 429.41 | 3.7098 | 4.3769 | 2.738 | 3.391 |
| 6 | 499.99 | 3.6567 | 4.5265 | 2.723 | 3.442 |
| 7 | 568.35 | 3.6030 | 4.6871 | 2.710 | 3.492 |
| 8 | 634.53 | 3.5488 | 4.8723 | 2.698 | 3.542 |
| 9 | 698.54 | 3.4944 | 5.0482 | 2.687 | 3.592 |
| 10 | 760.43 | 3.4393 | 5.2154 | 2.678 | 3.642 |
| 11 | 820.22 | 3.3835 | 5.4139 | 2.669 | 3.692 |
| **$(3)^4\Pi$** | | | | | |
| **v** | **$E_v$ ($cm^{-1}$)** | **$B_v \times 10^{-2}$ ($cm^{-1}$)** | **$D_v \times 10^{-8}$ ($cm^{-1}$)** | **$R_{min}$ (Å)** | **$R_{max}$ (Å)** |
| 0 | 64.05 | 4.2665 | 1.9158 | 2.833 | 2.983 |
| 1 | 190.80 | 4.2381 | 1.9564 | 2.785 | 3.047 |
| 2 | 315.64 | 4.2094 | 1.9982 | 2.755 | 3.095 |
| 3 | 438.59 | 4.1804 | 2.0382 | 2.731 | 3.136 |
| 4 | 559.65 | 4.1511 | 2.0833 | 2.711 | 3.174 |
| 5 | 678.83 | 4.1214 | 2.1259 | 2.694 | 3.209 |
| 6 | 796.13 | 4.0915 | 2.1734 | 2.679 | 3.242 |
| 7 | 911.54 | 4.0611 | 2.2156 | 2.666 | 3.275 |
| 8 | 1025.07 | 4.0304 | 2.2677 | 2.653 | 3.306 |
| 9 | 1136.72 | 3.9993 | 2.3123 | 2.642 | 3.337 |
| 10 | 1246.50 | 3.9678 | 2.3674 | 2.632 | 3.367 |
| 11 | 1354.38 | 3.9360 | 2.4157 | 2.622 | 3.397 |
| 12 | 1564.51 | 3.8710 | 2.5287 | 2.604 | 3.457 |
| 13 | 1666.74 | 3.8377 | 2.5867 | 2.596 | 3.486 |
| 14 | 1767.07 | 3.8041 | 2.6509 | 2.589 | 3.516 |
| 15 | 1865.50 | 3.7700 | 2.7098 | 2.581 | 3.545 |
| 16 | 1962.03 | 3.7353 | 2.7817 | 2.575 | 3.575 |
| 17 | 2056.64 | 3.7001 | 2.8513 | 2.568 | 3.605 |
| 18 | 2149.32 | 3.6644 | 2.9239 | 2.562 | 3.636 |
| 19 | 2240.08 | 3.6281 | 2.9999 | 2.556 | 3.666 |

**Table S2.** The rovibrational constants for the different vibrational levels of different electronic states of the pin-orbit coupling of CdBr molecule.

| $(1)^2\Pi_{3/2}$ | | | | | |
|---|---|---|---|---|---|
| v | $E_v$ ($cm^{-1}$) | $B_v \times 10^{-2}$ ($cm^{-1}$) | $D_v \times 10^{-8}$ ($cm^{-1}$) | $R_{min}$ (Å) | $R_{max}$ (Å) |
| 0 | 131.77 | 5.8804 | 1.2056 | 2.424 | 2.530 |
| 1 | 390.12 | 5.8415 | 1.1801 | 2.391 | 2.575 |
| 2 | 647.14 | 5.8025 | 1.1306 | 2.371 | 2.608 |
| 3 | 903.47 | 5.7595 | 1.0347 | 2.357 | 2.637 |
| 4 | 1160.29 | 5.7128 | 0.97329 | 2.346 | 2.663 |
| 5 | 1417.37 | 5.6666 | 0.99212 | 2.337 | 2.688 |
| 6 | 1673.49 | 5.6256 | 1.1044 | 2.328 | 2.711 |
| 7 | 1926.72 | 5.5911 | 1.1762 | 2.320 | 2.733 |
| 8 | 2176.42 | 5.5564 | 1.1274 | 2.313 | 2.754 |
| 9 | 2423.39 | 5.5196 | 1.1356 | 2.306 | 2.775 |
| 10 | 2667.61 | 5.4850 | 1.2004 | 2.299 | 2.795 |
| 11 | 2908.64 | 5.4515 | 1.1980 | 2.293 | 2.814 |
| 12 | 3146.67 | 5.4166 | 1.1796 | 2.287 | 2.833 |
| 13 | 3381.90 | 5.3812 | 1.1768 | 2.282 | 2.852 |
| 14 | 3614.33 | 5.3449 | 1.1486 | 2.278 | 2.871 |
| $(1)^4\Sigma^+_{1/2}$ | | | | | |
| v | $E_v$ ($cm^{-1}$) | $B_v \times 10^{-2}$ ($cm^{-1}$) | $D_v \times 10^{-8}$ ($cm^{-1}$) | $R_{min}$ (Å) | $R_{max}$ (Å) |
| 0 | 36.64 | 3.9247 | 4.6335 | 2.931 | 3.130 |
| 1 | 107.96 | 3.8623 | 4.9069 | 2.875 | 3.225 |
| 2 | 176.49 | 3.7986 | 5.1760 | 2.840 | 3.300 |
| 3 | 242.32 | 3.7350 | 5.3094 | 2.814 | 3.368 |
| 4 | 305.88 | 3.6771 | 5.0427 | 2.794 | 3.431 |
| 5 | 368.07 | 3.6292 | 4.8461 | 2.776 | 3.484 |
| 6 | 429.20 | 3.5748 | 6.0728 | 2.760 | 3.539 |
| 7 | 487.63 | 3.4962 | 7.4407 | 2.747 | 3.599 |
| 8 | 542.37 | 3.4182 | 7.2067 | 2.735 | 3.662 |
| 9 | 594.21 | 3.3419 | 7.9102 | 2.725 | 3.727 |
| 10 | 643.21 | 3.2699 | 7.2047 | 2.716 | 3.795 |
